# Quantitative CT evaluation after two cycles of induction chemotherapy to predict prognosis of patients with locally advanced oesophageal squamous cell carcinoma before undergoing definitive chemoradiotherapy/radiotherapy

**DOI:** 10.1007/s00330-022-08994-y

**Published:** 2022-08-04

**Authors:** Shuo Yan, Yan-Jie Shi, Chang Liu, Xiao-Ting Li, Bo Zhao, Yi-Yuan Wei, Lin Shen, Zhi-Hao Lu, Ying-Shi Sun

**Affiliations:** 1 Key Laboratory of Carcinogenesis and Translational Research (Ministry of Education/ Beijing), Department of Radiology, Peking University Cancer Hospital & Institute, No. 52, Fucheng Road, Hai Dian District, Beijing, 100142 China; 2 Key Laboratory of Carcinogenesis and Translational Research (Ministry of Education/ Beijing), Early Drug Development Center, Peking University Cancer Hospital & Institute, No. 52, Fucheng Road, Haidian District, Beijing, 100142 China; 3 Department of Gastrointestinal Oncology, Key Laboratory of Carcinogenesis and Translational Research (Ministry of Education/Beijing), Peking University Cancer Hospital & Institute, No. 52 Fu-Cheng Road, Hai-Dian District, Beijing, 100142 China

**Keywords:** Oesophageal squamous cell carcinoma, Tomography, X-ray computed, Prognosis, Response Evaluation Criteria in Solid Tumours

## Abstract

**Objective:**

To investigate the performance of quantitative CT analysis in predicting the prognosis of patients with locally advanced oesophageal squamous cell carcinoma (ESCC) after two cycles of induction chemotherapy before definitive chemoradiotherapy/radiotherapy.

**Methods:**

A total of 110 patients with locally advanced ESCC were retrospectively analysed. Baseline chest CT and CT after two cycles of induction chemotherapy were analysed. A multivariate Cox proportional-hazard regression model was used to identify independent prognostic markers for survival analysis. Then, a CT scoring system was established. Time-dependent receiver operating characteristic (ROC) curve analysis and the Kaplan-Meier method were employed for analysing the prognostic value of the CT scoring system.

**Results:**

Body mass index, treatment strategy, change ratios of thickness (ΔTH_max_), CT value of the primary tumour (ΔCTV_axial_) and the short diameter (ΔSD-LN), and the presence of an enlarged small lymph node (ESLN) after two cycles of chemotherapy were noted as independent factors for predicting overall survival (OS). The specificity of the presence of ESLN for death after 12 months was up to 100%. Areas under the curve value of the CT scoring system for predicting OS and progression-free survival (PFS) were higher than that of the RECIST (*p* < 0.05). Responders had significantly longer OS and PFS than non-responders.

**Conclusion:**

Quantitative CT analysis after two cycles of induction chemotherapy could predict the outcome of locally advanced ESCC patients treated with definitive chemoradiotherapy/radiotherapy. The CT scoring system could contribute to the development of an appropriate strategy for patients with locally advanced ESCC.

**Key Points:**

*• Quantitative CT evaluation after two cycles of induction chemotherapy can predict the long-term outcome of locally advanced oesophageal cancer treated with definitive chemoradiotherapy/radiotherapy.*

*• A CT scoring system provides valuable imaging support for indicating the prognosis at the early stage of therapy.*

*• Quantitative CT evaluation can assist clinicians in personalising treatment plans.*

**Supplementary Information:**

The online version contains supplementary material available at 10.1007/s00330-022-08994-y.

## Introduction

Oesophageal cancer is the eighth most common cancer and the seventh leading cause of cancer-related deaths worldwide [1]. Preoperative neoadjuvant chemotherapy followed by radical surgical resection is the first-line treatment strategy for locally advanced oesophageal squamous cell carcinoma (ESCC). One study showed that 13% of patients failed to achieve R0 resection even after preoperative neoadjuvant chemotherapy [2]. For patients who could not benefit from resection or those who could not tolerate surgery due to their poor clinical conditions, definitive chemoradiotherapy/radiotherapy (CRT/RT) is recommended as the alternative treatment [3]. However, over 50% of patients respond poorly to CRT/RT [4]. Concurrent molecular-targeted agents [5] and immune checkpoint inhibitor (ICI) therapy [5, 6] could be complementary strategies for improving tumour progression control in those patients. Timely diagnosis of patients who respond poorly to CRT/RT can assist clinicians in better adjusting therapeutic strategies and enable individualised and patient-specific treatment.

Changes in ESCC after induction therapy may reflect the biological behaviours of the tumour. Imaging modalities through assessing these changes may be advantageous to predict the response and prognosis of CRT/RT. Computed tomography (CT) is one of the common protocols to evaluate treatment response and prognosis of ESCC patients. The assessment of the changes in tumour burden during cancer therapeutics based on CT is done frequently through the Response Evaluation Criteria in Solid Tumours (RECIST, ver. 1.1) guideline. However, ESCC fails to meet the definition of target lesion in the RECIST (ver. 1.1) guideline, and the evaluation of ESCC is mainly based on metastatic lymph nodes. However, over 88% of metastatic lymph nodes of ESCC are smaller than 10 mm [7] and may be underestimated by the RECIST (ver. 1.1) guideline. A study reported that inter-criteria agreement between the RECIST and histological criteria was relatively low, and the RECIST (ver. 1.1) guideline was found inadequate for survival prediction [8].

Several studies have concentrated on CT measurement of the primary tumours, and they have revealed that changes after or during neoadjuvant therapy were strongly correlated to tumour regression grade in ESCC patients treated with neoadjuvant therapy and radical resection [9–11]. Other studies on lymph node status have shown that reduction of lymph node size after neoadjuvant therapy could predict histological responses of the tumour [11], and could be an independent prognostic factor for long-term survival after surgery [12]. To our knowledge, little research has concentrated on the changes in primary tumours and lymph node status using CT scans for survival analysis in patients treated with CRT/RT. The present study aimed to evaluate the performance of CT scan, based on the changes after two cycles of induction chemotherapy, to predict the survival of locally advanced ESCC patients treated with CRT/RT, and to contribute to the development of further effective treatment strategies for locally advanced ESCC.

## Methods

The study protocol was approved by the Ethics Committee of our hospital. Written informed consent was waived due to the retrospective nature of the study.

### Patients

**Fig. 1:**
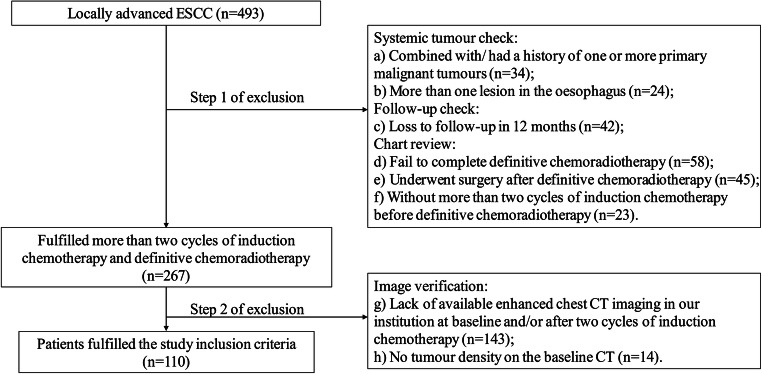
Flow diagram of eligible patients according to the inclusion and exclusion criteria

Data of 493 consecutive patients, who were firstly diagnosed with locally advanced ESCC between January 2014 and September 2019 in the department of gastrointestinal oncology of a single medical centre, were retrospectively analysed. Patients were excluded when they met the criteria shown in Fig. 1.

### CT protocols

All patients received contrast-enhanced multi-detector CT scanning of the chest before treatment (baseline CT) and after two cycles of induction chemotherapy (second CT). The CT scanning of the chest was performed using a 64-row helical CT scanner (Lightspeed VCT; General Electric Medical Systems) with the following parameters: tube voltage of 120 kVp, automatic current of 50–250 mA based on body weight, and detector collimation of 0.625 mm, with a helical pitch of 0.984. Maximum CT dose index was 53.45 mGy. After plain CT scanning, contrast-enhanced CT scanning was carried out 55 s after the beginning of injection of 80 mL non-ionic contrast medium Iohexol (Omnipaque 300; GE Healthcare) at a rate of 3.0 mL/s through the median cubital vein. Scan range covered from 2 cm above the lung apices to the adrenal glands at the end of inspiration in the supine position. Patients were asked to avoid eating and drinking for more than 4 h before CT scan. Axial CT images were reconstructed using a section width of 5.0 mm with the Advantage Workstation 4.4 software (GE Healthcare). Coronal and sagittal reformations were also reconstructed using a section width of 5.0 mm. Cervical and abdominal CT scans were performed for detecting distant metastases, and were acquired via standard scanning protocols (Supplementary Table 1).

### CT evaluation

The axial and reconstructed sagittal contrast-enhanced images of baseline CT and the second CT were reviewed repeatedly with an interval of 4 months by a radiologist (S.Y.) with 9 years of experience in chest imaging. The reviewer was blinded to patients’ survival outcomes. Measurements with a low consistency between the two reviews were excluded for further analysis. Quantitative measurements were determined by the average of the two reviews. Qualitative assessment was re-evaluated and confirmed by another radiologist (YJ. S.) with 18 years of experience in elimination of inconsistency.

**Fig. 2:**
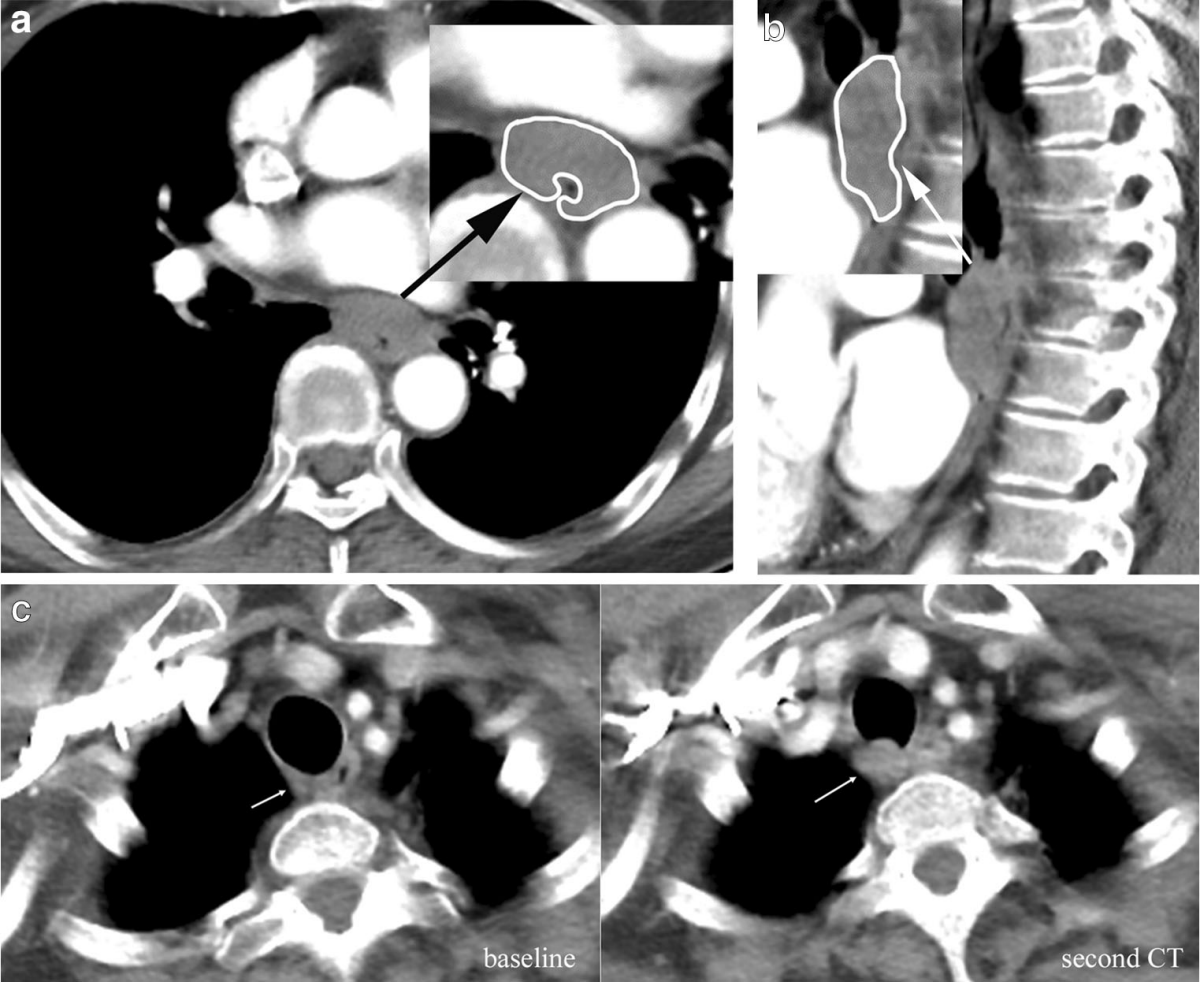
Regions of interest (ROIs). The ROIs (white circles) were manually drawn to encompass the tumour on axial image (**a**) on baseline CT and the second CT, and on sagittal image (**b**) avoiding necrosis, vessels, haemorrhage, and oesophageal lumen. **c** An enlarged small lymph node (ESLN) on the second CT. Baseline CT revealed a small lymph node with a short diameter of 4.4 mm in the right para-oesophageal region (white arrow), and CT after two cycles of induction chemotherapy showed an enlarged lymph node with a short diameter of 9.5 mm in the right para-oesophageal region (white arrow)

The maximum thickness (TH_max_) was measured perpendicularly to the lumen using the workstation’s electronic caliper on contrast-enhanced axial images in locations with a visible lumen. The regions of interest (ROIs) were manually delineated to encompass the tumour on the maximal section in axial and sagittal images avoiding necrosis, vessels, and oesophageal lumen (Fig. 2a, b). The maximum axial area (area_max-axial_) and average CT value of area_max-axial_ (CTV_axial_) of the tumour were obtained through ROIs of axial images. The maximum length (*L*_max_), maximum sagittal area (area_max-sag_), and average CT value of area_max-sag_ (CTV_sag_) were measured through ROIs of enhanced sagittal images. Baseline CT and the second CT measurements were conducted separately.

Intrathoracic and abdominal lymph nodes with a short-axis diameter larger than 10 mm and supraclavicular lymph nodes with a size of larger than 5 mm or heterogeneously enhanced with central necrosis on baseline CT were considered CT metastatic lymph nodes (CTLN) [13]. The short axial diameter of the largest CTLN (SD-LN) on baseline CT was measured on contrast-enhanced axial images. The ROI was manually delineated to encompass the LN on the maximal section in axial images avoiding necrosis. The average CT value of the maximum area (CTV-LN) was obtained through the ROIs of the largest LN. Evaluation of the second CT was conducted with reference to the baseline CT data for the same lymph nodes. If there was no CTLN on the baseline CT, it was recorded as 0 mm for both CT scans. Enlarged small lymph nodes (ESLN) on the second CT scan were also recorded. An ESLN was defined as a lymph node less than 10 mm in short diameter at both baseline and second CT, with an increase ≥ 2 mm or 20% in short diameter after induction chemotherapy at the second CT compared with baseline CT (Fig. 2c), or a new lymph node on the second CT. A new lymph node was defined as the presence of a new small lymph node with a diameter larger than 5 mm and less than 10 mm in the second CT when this lymph node was not observed in the baseline CT. The regional lymph nodes in ESCC included lymph nodes from the supraclavicular region to the level of the coeliac trunk (listed in Supplementary Table 2) [14]. More remote nonregional lymph nodes, such as the cardio phrenic region or the posterior thoracic paraaortic region, equated to distant metastatic disease and were not included in our study.

Quantitative measurements were recorded as change ratios (Δmeasurement), which were calculated as follows:

$$ \Delta \mathrm{measurement}=\frac{\left(\mathrm{measurement}\ \mathrm{on}\ \mathrm{the}\ \mathrm{second}\ \mathrm{CT}-\mathrm{measurement}\ \mathrm{on}\ \mathrm{the}\ \mathrm{baseline}\ \mathrm{CT}\right)}{\mathrm{measurement}\ \mathrm{on}\ \mathrm{the}\ \mathrm{baseline}\ \mathrm{CT}}. $$

Change ratios of TH_max_ (ΔTH_max_), area_max-axial_ (Δarea_max-axial_), CTV axial (ΔCTV axial), *L*_max_ (Δ*L*_max_), area_max-sag_ (Δarea_max-sag_), CTV sag (ΔCTV sag), SD-LN (ΔSD-LN), and CTV-LN (ΔCTV-LN) were obtained by the above-mentioned formula.

### Clinical evaluation

Patients underwent gastrointestinal endoscopy and cervical and abdominal contrast-enhanced CT within 2 weeks before or after baseline CT of the chest. Tumour staging was performed according to the eighth edition of the Union for International Cancer Control–American Joint Committee on Cancer (UICC-AJCC) tumour, node, metastasis (TNM) staging system for ESCC.

Body mass index (BMI) at baseline was calculated based on height and weight at the first treatment. Differentiation of tumours was pathologically confirmed on biopsy specimens obtained by gastrointestinal endoscopy.

Evaluation of tumours’ responses based on the RECIST (ver. 1.1) guideline [15] was conducted by a radiologist (YJ. S.) with 18 years of experience in chest imaging. According to the RECIST (ver. 1.1) guideline, oesophageal tumours were measured as non-target lesions and were qualitatively followed as “present”, “absent” or “progressive disease (PD)”. Lymph nodes with a short diameter larger than 15 mm were recorded as target lesions. Partial response (PR) was noted when the sum of the diameters of target lymph nodes had a reduction of ≥ 30% and persistence of tumour. PD was recognised when there was an increase of ≥ 20% in the sum diameter of target lymph nodes in patients who suffered from new lesions, or there was an unequivocal progression in non-target tumours. Stable disease (SD) was defined as neither sufficient shrinkage to qualify for PR nor sufficient increase to qualify for PD.

### Treatment strategies

There were three options for induction chemotherapy: (a) paclitaxel combined with cisplatin; (b) paclitaxel combined with carboplatin; and (c) paclitaxel alone for elderly patients or patients with poor systemic conditions. In total, 61 (55.5%) patients received concurrent CRT with a dose of 50.4 Gy after 2–6 cycles of induction chemotherapy, and the remaining 49 (44.5%) patients received RT alone at a dose of 50.4 Gy after 2–6 cycles of induction chemotherapy.

### Follow-up

All the patients were followed up regularly during and after treatment. Follow-up assessment consisted of outpatient interviews at a 3-month interval for 1 year, followed by a 6-month interval for 2 years and finally a 12-month interval until death. Systemic physical examination and cervical, thoracic and abdominal CT examinations were performed during follow-ups. The date of first chemotherapy, the last follow-up, oesophageal cancer-specific death, the cause of death and date of disease progression based on systemic evaluation were recorded. Overall survival (OS) and progression-free survival (PFS) were measured from the first chemotherapy until oesophageal cancer-specific death and disease progression. For patients who were alive or without disease progression, the OS and PFS were censored at the last follow-up visit.

### Statistical analysis

Intraclass correlation (ICC) efficiency was calculated to evaluate the inter-review agreement for measurements. An ICC ≥ 0.70 indicated a good consistency and an ICC < 0.70 reflected a poor consistency. A multivariate Cox proportional-hazard regression model with stepwise comparisons was used to identify independent prognostic markers for death and to obtain hazard ratios (HRs). A CT scoring system was built based on the *β* coefficient from Cox regression model according to the method brought by Sullivan [16]. Time-dependent receiver operating characteristic (ROC) curve analysis was applied to confirm the diagnostic ability of the CT scoring system and the RECIST (ver. 1.1). The Kaplan-Meier method and the log-rank test were utilised to compare survival curves of responders and non-responders that were grouped based on the CT scoring system and the RECIST (ver. 1.1).

The statistical analysis was carried out using the SPSS 22.0 (IBM) and SAS 9.4 (SAS Institute Inc.) software. *p* < 0.05 was considered statistically significant.

## Results

### Patients’ clinical characteristics

A total of 110 patients with locally advanced ESCC were enrolled, including 98 (89.1%) male and 12 (10.9%) female patients, with an average age of 59.9 ± 7.8 years old. The BMI of enrolled patients, clinical TNM (cTNM) staging, differentiation, and location of tumours are summarised in Table 1.

**Table 1:** Patients’ demographic data

Characteristic	Case (%)
Male	98 (89.1)
Female	12 (10.9)
Age (years old)	(59.9 ± 7.8)
BMI (kg/m^2^)	(22.49 ± 3.39)
cTNM staging	
Stage II	5 (4.5)
Stage III	73 (66.4)
Stage IV	32 (29.1)
Differentiation	
Well	16 (14.5)
Moderate	55 (50.0)
Poor	39 (35.5)
Location of tumour	
Cervical	7 (6.4)
Upper thoracic	31 (28.2)
Middle thoracic	49 (44.5)
Lower thoracic	18 (16.4)
Abdominal	5 (4.5)

*BMI* body mass index, *cTNM staging* clinical tumour-node-metastasis staging

Median follow-up time was 20.1 (range, 4.3~78.8) months. Besides, 48 (43.6%) patients were alive and had censored data until the data analysis was performed. The median follow-up time for alive patients was 30.8 (range, 15.4~78.1) months. During the follow-up period, 83 (75.5%) patients had disease progression. The median survival time and time to progression were 28.6 (95% confidence interval (CI), 21.5–35.7) months and 13.1 (95% CI, 10.1–16.0) months, respectively.

### CT measurements and correlation with long-term survival

Analysis of ICC on CT measurements showed that ICCs for Δarea_max-sag_ and ΔCTV_sag_ were 0.44 and 0.50 (ICCs < 0.70), while those for ΔTH_max_, Δarea_max-axial_, ΔCTV_axial_, Δ*L*_max_, ΔSD-LN, and ΔCTV-LN were 0.82, 0.75, 0.74, 0.70, 0.92, and 0.80 (all ICCs ≥ 0.70), respectively. ΔArea_max-sag_ and ΔCTV_sag_ were excluded due to the poor consistency between the two measures.

The results of Cox regression analysis are listed in Table 2. It revealed that the BMI and followed treatment strategy after induction chemotherapy were independent factors that were correlated with OS. In addition, ΔTH_max_, ΔCTV_axial_, and ΔSD-LN were independent factors that were positively correlated with OS. The presence of ESLN was noted as an independent factor that was correlated with an increased risk of OS.

**Table 2:** Univariate and multivariate Cox regression analyses of clinical, pathological, and CT findings according to the overall survival

	Univariate analysis		Multivariate analysis		
	HR (95% CI)	*p*	*β*	HR (95% CI)	*p*
Age (years old)	0.97 (0.94–1.00)	0.051			
Gender		0.696			
Male	Reference				
Female	0.85 (0.36–1.97)				
BMI (kg/m^2^)	0.90 (0.83–0.98)	0.013*	− 0.12	0.89 (0.82–0.97)	0.005*
Location of tumour		0.035*			
Cervical	Reference				
Upper thoracic	0.50 (1.7–1.51)				
Middle thoracic	0.66 (0.23–1.91)				
Lower thoracic	1.57 (0.52–4.80)				
Abdominal	1.36 (0.30–6.11)				
Differentiation		0.359			
Well	Reference				
Moderate	1.69 (0.78–3.67)	0.263			
Poor	1.32 (0.58–3.01)	0.196			
cTNM stage		0.649			
II	Reference				
III	1.32 (0.41–4.27)				
IV	1.04 (0.30–3.60)				
Followed treatment protocol		0.001*	0.88		0.002*
CRT	Reference			Reference	
RT	2.32 (1.38–3.88)			2.40 (1.40–4.13)	
ΔTH_max_	14.20 (4.20–48.13)	0.000*	2.55	12.81 (3.31–49.60)	0.000*
ΔArea_max-axial_	3.77 (1.67–8.50)	0.001*			
ΔCTV_axial_	1.96 (1.37–2.81)	0.000*	0.43	1.53 (1.01–2.33)	0.045*
Δ*L*_max_	3.48 (0.95–12.70)	0.060			
ΔSD-LN	12.34 (3.83–39.75)	0.000*	2.00	7.37 (2.06–26.40)	0.002*
ΔCTV-LN	1.07 (1.00–1.14)	0.038*			
ESLN		0.009*	1.70		0.002*
None	Reference			Reference	
Present	3.48 (1.37–8.81)			5.44 (1.90–15.56)	

All the lymph nodes included in the evaluation were located in the region range from the supraclavicular region to the level of the coeliac trunk. ΔMeasurement = (measurement on the second CT − measurement on the baseline CT) / measurement on the baseline CT

*HR* hazard ratio, *CI* confidence interval, *BMI* body mass index, *CRT* chemoradiotherapy, *RT* radiotherapy, *ΔTH*_*max*_ change ratios in maximum thickness of tumour, *ΔArea*_*max-axial*_ change ratios in maximum axial area of tumour, *ΔCTV*_*axial*_ change ratios in average CT value of maximum axial area of tumour, *ΔL*_*max*_ change ratios in the maximum length of tumour, *ΔSD-LN* change ratios in short axial diameter of largest lymph node, *ΔCTV-LN* change ratios in average CT value of maximum area of lymph nodes, *ESLNs* enlarged small lymph nodes

*Marked as *p* < 0.05

It was found that among 8 patients with ESLN, 7 (87.5%) patients died in 6 months, and the other patient died in 12 months. Among them, 4 (50%) patients had decreased TH_max_, CTV_axial_, and SD-LN on the second CT, 3 (37.5%) patients had increased CTV_axial_, and the other one had increased SD-LN.

### CT scoring system and its predictive value compared with RECIST (ver. 1.1)

**Fig. 3:**
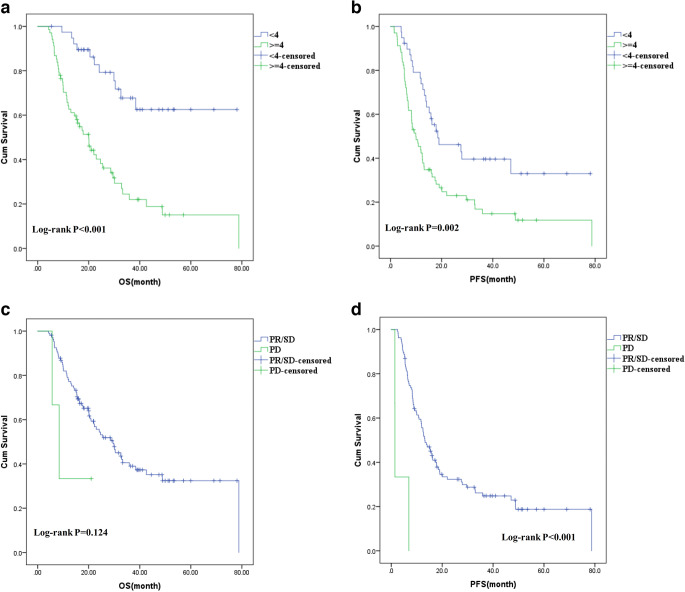
(Coloured) Kaplan-Meier curves for overall survival (**a**, **c**) and progression-free survival (**b**, **d**) in responders and non-responders that were grouped based on the CT scoring system and the RECIST (ver. 1.1). Curves were compared using the log-rank test

A CT scoring system was established (Table 3). Cut-off values of CT measurements were determined by the time-dependent ROC curve analysis, quartiles, and stepwise analysis. The final score was calculated by the sum of these scores. Patients with CT score < 4 were marked as responders and those with CT score ≥ 4 were marked as non-responders. Responders showed significantly longer OS and PFS than non-responders (Fig. 3a, b). Besides, the 3-year survival rate for non-responders was 21.3%, which was significantly lower than that for responders (68.8%, *p* < 0.001). In addition, the 3-year PFS for non-responders was 14.3%, which was significantly lower than that for responders (47.6%, *p* = 0.002). The estimated mean survival time for responders and non-responders was 57.6 (range, 48.2~67.0) months and 27.6 (range, 21.0~34.2) months, respectively.

**Table 3:** The CT scoring system for the prognosis prediction

Evaluations		Score
Followed treatment protocol	CRT	0
	RT	1
BMI (kg/m^2^)	< 18.5	2
	18.5 to 24	1
	> 24	0
ΔTH_max_	< − 0.25	0
	≥ − 0.25	1
ΔCTV_axial_	< − 0.5	0
	− 0.5 to − 0.1	2
	> − 0.1	3
ΔSD-LN	< − 0.1	0
	≥ − 0.1	1
ESLN	No	0
	Yes	2

Δmeasurement = (measurement on the second CT − measurement on the baseline CT) / measurement on the baseline CT

*BMI* body mass index, *CRT* chemoradiotherapy, *RT* radiotherapy, *ΔTH*_*max*_ change ratio in maximum thickness of tumour, *ΔCTV*_*axial*_ change ratio in average CT value of maximum axial area of tumour, *ΔSD-LN* change ratio in short axial diameter of largest metastatic lymph node, *ESLN* enlarged small lymph nodes

Among the 110 patients, 51 (46.4%) patients were evaluated as PR based on RECIST (ver. 1.1), 56 (50.9%) patients as SD, and 3 (2.7%) patients as PD. Among patients with PD, 2 patients had an unequivocal progression in primary tumours accompanied by progression in target lymph nodes, and the other one showed progression in target lymph nodes. Patients with PR/SD showed no statistical difference in OS compared to patients with PD (Fig. 3c). Patients with PR/SD showed significantly longer PFS than those with PD (Fig. 3d). Besides, the 3-year survival rate for PD was 0.0%, which was not significantly different from that for PR/SD (39.0%, *p* = 0.285). The estimated mean survival time for PR/SD and PD was 39.2 (range, 32.9~45.5) months and 11.7 (range, 4.2~19.2) months, respectively. Moreover, the 3-year PFS rate for PD was 0.0%, which was not significantly different from that for PR/SD (24.8%, *p* = 0.441).

The AUC values of the CT scoring system for 6-month, 1-year, 3-year, and 5-year OS were significantly higher than those of the RECIST (ver. 1.1) (*p* < 0.05) (Table 4). The AUC values of the CT scoring system for 1-year and 3-year PFS were significantly higher than those of the RECIST (ver. 1.1) (*p* < 0.05) (Table 4).

**Table 4:** Comparison of the performance of the CT scoring system and the RECIST (ver. 1.1) for predicting overall survival and progression-free survival

	Time	Criteria	Sensitivity	Specificity	AUC (95%CI)	*p*
OS	6 months	CT scoring system	1.000	0.885	0.970 (0.954–0.986)	< 0.007*
		RECIST v1.1	0.799	0.471	0.591 (0.335, 0.846)	
	1 year	CT scoring system	0.798	0.732	0.871 (0.788–0.955)	0.001*
		RECIST v1.1	0.840	0.561	0.534 (0.447, 0.621)	
	3 years	CT scoring system	0.614	0.92	0.831 (0.746–0.916)	< 0.001*
		RECIST v1.1	0.625	0.680	0.515 (0.480, 0.550)	
	5 years	CT scoring system	0.982	0.750	0.835 (0.628–0.999)	0.005*
		RECIST v1.1	0.597	0.5	0.513 (0.482, 0.545)	
PFS	6 months	CT scoring system	0.546	0.943	0.778 (0.664–0.892)	0.093
		RECIST v1.1	0.818	0.529	0.540 (0.440–0.639)	
	1 year	CT scoring system	0.619	0.793	0.767 (0.687–0.846)	0.019*
		RECIST v1.1	0.760	0.655	0.530 (0.474, 0.586)	
	3 year	CT scoring system	0.499	0.947	0.776 (0.684–0.867)	0.029*
		RECIST v1.1	0.650	0.789	0.518 (0.480, 0.556)	
	5 years	CT scoring system	0.915	0.667	0.734 (0.369–0.999)	0.343
		RECIST v1.1	0.628	0.667	0.517 (0.482, 0.550)	

*OS* overall survival, *PFS* progression-free survival, *AUC* area under curve, *RECIST* Response Evaluation Criteria in Solid Tumours

*Marked as *p* < 0.05

## Discussion

In the present study, we concentrate on the predictive value of quantitative CT measurements after 2 cycles of induction chemotherapy for long-term prognosis in locally advanced ESCC patients treated with definitive CRT/RT. The results revealed that CT measurements of primary tumours and lymph nodes after 2 cycles of induction chemotherapy were independent predictive factors for long-term outcomes. A CT scoring system established based on CT measurements showed a better prognostic value than the RECIST (ver. 1.1).

Our study indicated that reduction in maximum thickness of primary tumours after 2 cycles of induction chemotherapy before CRT/RT was an independent predictor that was associated with OS of locally advanced ESCC patients treated with CRT/RT. The results are consistent with previous studies on ESCC patients treated with neoadjuvant therapy followed by radical surgical resection. Previous studies reported that the tumour thickness was correlated with tumour regression grade of resected tumour and OS, and it could be an independent predictive factor for pathological complete response [9, 10, 17–19]. Urakawa et al showed that lymph node response to neoadjuvant therapy predicted long-term survival more precisely than the primary response in patients with metastatic ESCC [11]. Our study also provided strong evidence that reduction in the short diameter of the largest CT metastatic lymph nodes was an independent predictor for OS in locally advanced ESCC patients treated with CRT/RT. However, the difference between our study and Urakawa et al’s study is that the HR of ΔTH_max_ from the Cox regression analysis is higher than that of changes in the diameter of lymph nodes in our study. We speculate that the changes of the largest metastatic lymph nodes may not be better than the primary tumours in predicting the long-term outcome in locally advanced ESCC patients treated with CRT/RT.

Change of CT enhancement of the primary tumour was another feature of primary tumours that was independently correlated with OS in our study. This is consistent with the roles of CT enhancement in tumour response and survival analysis in ESCC patients treated with neoadjuvant therapy [9, 20]. The enhancement reflects the increased microvascular circulation and highly permeable neovascularisation; thus, the reduction of the contrast-enhanced CT value in post-treatment ESCC could be reasonably speculated as a decrease of active tumour components, leading to a prolonged survival.

Our study also showed that the decrease in tumour length was not correlated with survival. It revealed that during measurement, the ESCC tumour tended to initially shrink in thickness in lieu of the length during chemotherapy. Previous studies reported that tumour length on pre-treatment images was a prognostic factor for survival [21, 22]. Therefore, we suggest that clinicians should pay further attention to the tumour length on pre-treatment images rather than the changes in the length during treatment. To ensure a good reproducibility, measurements with poor consistency, tumour area, and contrast-enhanced CT value on sagittal images were excluded from the analysis. The oedema after induction chemotherapy, which made it difficult for us to accurately determine the boundaries of the tumour on sagittal images, may be the leading cause of the measurement inconsistency.

In addition, we assessed the changes in small lymph nodes, and the results suggested that the presence of ESLN after two cycles of induction chemotherapy was an independent factor that was correlated with OS. To our knowledge, this is the first study that considered small lymph nodes in the prognostic analysis of ESCC patients. We focused on small lymph nodes with short diameters less than 10 mm because of the underestimated evaluation of small lymph nodes in oesophageal cancer. In RECIST 1.1 criteria, lymph nodes with diameters larger than 10 mm and less than 15 mm are classified as non-target lesions at baseline CT imaging. The small lymph nodes with diameters less than 10 mm on baseline CT, which increase to larger than 10 mm on second CT, are considered new lesions [15]. The lymph nodes larger than 10 mm at either baseline or second CT could be assessed according to RECIST 1.1 criteria. However, the lymph nodes with diameters less than 10 mm in both baseline and second CT have not been assessed according to RECIST 1.1 criteria in clinical practice. Although 10 mm in short diameter is commonly used as a threshold for CT to indicate whether it is a lymph node metastasis or not, its accuracy is still questionable. The presence of ESLN in CT imaging is an indicator of lymph node involvement, accompanied by low sensitivity (12–35%) [7, 23]. In this study, we investigated whether the small lymph nodes could affect the prognosis in ESCC patients. The result showed that 50% of patients with ESLN were not accompanied by progression of primary tumours or CT metastatic lymph nodes, suggesting that ESLN is of great significance as an independent factor for response evaluation. We strongly suggest that changes in lymph nodes smaller than 10 mm should be taken into account in response evaluation.

A practical CT scoring system combining the changes of primary tumours and lymph nodes revealed a better predictive capability for OS and PFS compared with RECIST (ver. 1.1). We speculate that the CT-scoring system is a notable alternative criterion for PFS and OS determination in patients with ESCC treated with RT/CRT. In the present study, the majority of patients (107/110) were responders based on the assessment by RECIST (ver1.1). Among them, 65 patients were non-responders according to the assessment of the CT-scoring system. We suggest that further effective treatment strategies should be developed for improving the control of tumour progression for these patients. Therapeutic strategy after two cycles of induction chemotherapy was included in the analysis as a covariate factor in this retrospective study. Both uni- and multivariate Cox analyses showed that the followed strategy was one of the factors that independently affected the OS for ESCC patients. RT was associated with a 2.4-fold increased risk of death compared to CRT. The result is consistent with that of a previous study [24]. So, the followed RT/CRT strategy after two cycles of induction chemotherapy was added to the CT scoring system to ensure that the scoring system can assess the prognosis of patients receiving both CRT and RT. Differences in final treatment regimens would lead to differences in CT scores. When the patient could not tolerate CRT and is planning to be treated with RT, the CT score increases by 1 point. When the total score is ≥ 4 points, it indicates that the long-term prognosis of the patient is not satisfying. Complementary strategies would be needed to improve tumour control in those patients. When the patient plans to receive CRT, the CT score does not need to be upregulated. Our findings may lead to the establishment of personalised therapy for patients with locally advanced ESCC.

Our study has several limitations. Firstly, this was a single-centre, retrospective study with a small sample size, and selection bias might have influenced the results. However, the study population was drawn from a consecutive database and strict inclusion and exclusion criteria were designed; thus, the study population was still representative. We are looking forward to prospective cohorts from different centres to validate the proposed methodology. Secondly, no internal or external validation has been conducted in this study because of the limited sample size. Thus, prospective multi-centre validation is required to generalise our findings. Thirdly, oesophageal adenocarcinoma (EA) has been excluded from our study because of the differences in genomic and molecular characterisations, responses to chemoradiotherapy, and treatment protocols between EA and ESCC [25–27]. The evaluation system for assessing the response of EA should be considered in another study.

In conclusion, the present study proves that CT quantitative analysis after 2 cycles of induction chemotherapy could predict long-term outcomes of locally advanced ESCC patients treated with CRT/RT. A CT scoring system can be valuable imaging support for the prognosis indication at the early stage of therapy, and it can assist clinicians in personalising treatment plans.

## Supplementary Information

ESM 1 (PDF 87 kb)

## Abbreviations

CTV-LN: Average CT value of the maximum area of the largest CT metastatic lymph nodes
CTV_axial_: Average CT value of maximum axial area
CTV_sag_: Average CT value of maximum sagittal area
BMI: Body mass index
CRT/RT: Chemoradiotherapy/radiotherapy
cTNM: Clinical tumour, node, metastasis
CR: Complete response
CI: Confidence interval
CTLN: CT metastatic lymph nodes
ESLN: Enlarged small lymph node
HR: Hazard ratio
ICC: Intraclass correlation efficient
Area_max-axial_: Maximum axial area
*L*_max_: Maximum length
Area_max-sag_: Maximum sagittal area
TH_max_: Maximum thickness
EA: Oesophageal adenocarcinoma
ESCC: Oesophageal squamous cell carcinoma
OS: Overall survival
PR: Partial response
PFS: Progression-free survival
PD: Progressive disease
RECIST: Response Evaluation Criteria in Solid Tumours
SD-LN: Short axial diameter of the largest CT metastatic lymph nodes
SD: Stable disease
TNM: Tumour, node, metastasis

## Declarations

### Guarantor

The scientific guarantor of this publication is Ying-Shi Sun.

### Statistics and biometry

No complex statistical methods were necessary for this paper.

### Methodology

• retrospective

• diagnostic or prognostic study

• performed at one institution
