# Supplementary material for: Quantitative CT evaluation after two cycles of induction chemotherapy to predict prognosis of patients with locally advanced oesophageal squamous cell carcinoma before undergoing definitive chemoradiotherapy/radiotherapy
Source: Eur Radiol. 2022 Aug 4;33(1):380–90. doi: 10.1007/s00330-022-08994-y (PMC9755097; doi:10.1007/s00330-022-08994-y)
Supplement: Supplementary file 1 — (PDF 87 kb) [file 330_2022_8994_MOESM1_ESM.pdf]

Supplementary Table 1 Scanning protocols for cervical and abdominal CT

	Cervical CT	Abdominal CT
Body position	Supine, arms at the side of the body	Supine, arms at the side of the head
Tube current	Automatic based on body weight (120-300mA)	Automatic based on body weight (120-400mA)
Tube voltage	120 kVp	120 kVp
Detector collimation	0.625mm	0.625mm
Helical pitch	0.984	0.984
Contrast enhanced phases (time from injection to the scan)	Arterial phase (28s)<br>Venous phase (55s)	Arterial phase (35s)<br>Portal venous phase (60s)<br>Delayed phase (120s)
Rate of injection	2.5-3.0 mL/s	3 mL/s

Supplementary Table 2 Regional lymph nodes of oesophageal cancer according to the 8th edition of the AJCC/UICC cancer staging manuals [1]

	Stations of lymph nodes
1R	right lower cervical paratracheal nodes
1L	left lower cervical paratracheal nodes
2R	right upper paratracheal nodes
2L	left upper paratracheal nodes
4R	right lower paratracheal nodes
4L	left lower paratracheal nodes
7	subcarinal nodes
8U	Upper thoracic paraesophageal nodes
8M	Middle thoracic paraesophageal lymph nodes
8Lo	Lower thoracic paraesophageal lymph nodes
9R/L	Pulmonary ligament node
15	Diaphragmatic nodes
16	Paracardial nodes
17	Left gastric nodes
18	Common hepatic nodes
19	Splenic nodes
20	Celiac nodes

1 Rice TW, Ishwaran H, Ferguson MK, Blackstone EH, Goldstraw P (2017) Cancer of the esophagus and esophagogastric junction: an eighth edition staging primer. J Thorac Oncol 12(1):36-42
